# Non-Invasive Estimation of Intracranial Pressure-Derived Cerebrovascular Reactivity Using Near-Infrared Spectroscopy Sensor Technology in Acute Neural Injury: A Time-Series Analysis

**DOI:** 10.3390/s24020499

**Published:** 2024-01-13

**Authors:** Alwyn Gomez, Logan Froese, Tobias J. G. Bergmann, Amanjyot Singh Sainbhi, Nuray Vakitbilir, Abrar Islam, Kevin Y. Stein, Izabella Marquez, Younis Ibrahim, Frederick A. Zeiler

**Affiliations:** 1 Section of Neurosurgery, Department of Surgery, Rady Faculty of Health Sciences, University of Manitoba, Winnipeg, MB R3E 0W2, Canada; frederick.zeiler@umanitoba.ca; 2 Department of Human Anatomy and Cell Science, Rady Faculty of Health Sciences, University of Manitoba, Winnipeg, MB R3E 0W2, Canada; 3 Department of Biomedical Engineering, Price Faculty of Engineering, University of Manitoba, Winnipeg, MB R3T 5V6, Canada; log.froese@gmail.com (L.F.); amanjyot.s.sainbhi@gmail.com (A.S.S.); vakitbir@myumanitoba.ca (N.V.); islama9@myumanitoba.ca (A.I.); steink34@myumanitoba.ca (K.Y.S.); younis.ibrahim@umanitoba.ca (Y.I.); 4 Department of Biosystems Engineering, Price Faculty of Engineering, University of Manitoba, Winnipeg, MB R3T 5V6, Canada; bergmant@myumanitoba.ca (T.J.G.B.); marquezi@myumanitoba.ca (I.M.); 5 Undergraduate Medicine, Rady Faculty of Health Sciences, University of Manitoba, Winnipeg, MB R3E 0W2, Canada; 6 Centre on Aging, University of Manitoba, Winnipeg, MB R3E 0W2, Canada; 7 Division of Anaesthesia, Department of Medicine, Addenbrooke’s Hospital, University of Cambridge, Cambridge CB2 0QQ, UK; 8 Department of Clinical Neurosciences, Karolinksa Institutet, 171 77 Stockholm, Sweden

**Keywords:** cerebrovascular reactivity monitoring, intracranial pressure monitoring, near-infrared spectroscopy, neurotrauma

## Abstract

The contemporary monitoring of cerebrovascular reactivity (CVR) relies on invasive intracranial pressure (ICP) monitoring which limits its application. Interest is shifting towards near-infrared spectroscopic regional cerebral oxygen saturation (rSO_2_)-based indices of CVR which are less invasive and have improved spatial resolution. This study aims to examine and model the relationship between ICP and rSO_2_-based indices of CVR. Through a retrospective cohort study of prospectively collected physiologic data in moderate to severe traumatic brain injury (TBI) patients, linear mixed effects modeling techniques, augmented with time-series analysis, were utilized to evaluate the ability of rSO_2_-based indices of CVR to model ICP-based indices. It was found that rSO_2_-based indices of CVR had a statistically significant linear relationship with ICP-based indices, even when the hierarchical and autocorrelative nature of the data was accounted for. This strengthens the body of literature indicating the validity of rSO_2_-based indices of CVR and potential greatly expands the scope of CVR monitoring.

## 1. Introduction

Cerebrovascular reactivity (CVR) is the brain’s ability to maintain a stable cerebral blood flow (CBF), through vasoconstriction and vasodilation, over a range of arterial blood pressures (ABP) or cerebral perfusion pressures (CPP) [1,2]. Following moderate to severe acute biomechanical injury, termed traumatic brain injury (TBI), dysfunctional CVR has been identified as a contributor to ongoing brain injury throughout the acute injury phase. While there are various neuroimaging-based methods that leverage single photon emission computerized tomography (SPECT), positron emission tomography (PET), and magnetic resonance imaging (MRI), these methods are not suitable in the acute phase of brain injury. They are cumbersome and require the transportation of the critically ill patient. Additionally, by their nature, they provide a measure of CVR at a moment in time and require manually modulating ABP, which might not be tolerated in a critically ill patient [3]. Contemporary methods of continuously monitoring CVR operate through a continuously updating correlation coefficient between slow-wave vasogenic fluctuations in a surrogate for CBF/cerebral blood volume (CBV) and a surrogate of driving pressure, typically ABP or CPP [4,5,6].

The most well studied and implemented of these CVR indices is the pressured reactivity index (PRx) [7,8,9,10,11,12,13,14]. PRx utilizes intracranial pressure (ICP) as a surrogate for CBV and ABP as a surrogate for driving pressure [6]. ICP is typically monitored through either an intraparenchymal probe (strain-gage or fiberoptic) or through a catheter placed in a cerebral ventricle. Both methods require drilling into the patient’s skull and placement within the brain. In the case of an ICP probe, the sensor is placed into brain tissue of the patient while a ventricular catheter placement is made in the lateral ventricle of the brain where pressure in the fluid space can then be transduced. As ICP monitoring is often indicated in the critical care management of TBI patients, its use as a surrogate for CBV is reasonable. However, the reliance of PRx on invasively derived ICP means that it is not available in settings in which ICP monitoring is not already indicated or available. As invasive ICP monitoring is mostly only clinically indicated in the early phases of moderate to severe brain injury, lower grade brain injuries and phases of brain injury beyond the early phases of injury are not amenable to this form of monitoring. Additionally, by the nature of intracranial pressure–volume relationships, ICP can only act as a global surrogate for CBV and, as such, has limited spatial resolution. This has led to interest in alternative methods of measuring CVR that are less invasive and have improved spatial resolution [6].

The cerebral oximetry indices (COx and COx_a) have been proposed as a possible alternative to PRx. They leverage near-infrared spectroscopic (NIRS) regional cerebral oxygen saturation (rSO_2_) as a surrogate for CBV and CPP as a surrogate for driving pressure in the case of COx and ABP in the case of COx_a [15,16]. Briefly, NIRS-based cerebral oximetry works by leveraging the ability of near-infrared light to penetrate through the skull and scalp and into the brain parenchyma. In the parenchyma, it is scattered or absorbed though the chromophore hemoglobin in both its oxygenated (OxHgB) or deoxygenated (deOxHgB) forms. By utilizing the modified Beer–Lambert law, the relative concentrations of OxHgB and deOxHgB in the cerebral microvasculature can be derived and the rSO_2_ calculated. The details of NIRS-based cerebral oximetry are beyond the scope of this paper, but the interested reader should be directed to recent reviews on the topic [17,18,19].

The rSO_2_-based indices of CVR have shown promise and are the only indices, aside from PRx, to have been shown to be able to detect the lower limit of cerebrovascular reactivity in large animal models [15,16]. Recent work from our group has also shown that they cluster with PRx in multidimensional physiologic space and that ICP and rSO_2_ respond similarly to a sudden impulse in ABP [20]. However, prior to the widespread adoption of COx and COx_a, their relationship to PRx must be better quantified and modeled.

The aim of this study is to examine if PRx can be modeled by COx and COx_a through leveraging linear mixed effects modeling and time-series analysis in a cohort of moderate and severe TBI patients. This will help uncover more information on how these indices relate to one another.

## 2. Materials and Methods

### 2.1. Study Design

A single centre retrospective cohort study was performed leveraging the Winnipeg Acute TBI Database. Prospectively collected high-resolution physiologic data were collected from adult moderate to severe TBI patients admitted to the Winnipeg Health Sciences Centre Intensive Care Unit (ICU). Only subjects with invasive ICP and ABP monitoring, as well as concurrent NIRS-based rSO_2_, were included. Data were collected between April 2019 and December 2022. All patients were cared for utilizing current ICP- and CPP-based Brain Trauma Foundation guidelines [21]. Of note, both rSO_2_ as a raw parameter and continuous CVR metrics were not considered in patient care.

### 2.2. Ethical Considerations

All data were collected following full approval by the Health Sciences Centre Research Impact Committee and the University of Manitoba Biomedical Research Ethics Board (H2017:181, H2017:188, B2018:103, H2020:118, B2023:001).

### 2.3. Data Collection

ABP and ICP, as well as left and right rSO_2_, were collected as high-resolution data streams. ABP was invasively measured utilizing radial arterial lines, zeroed at the level of the tragus. ICP was monitored using intra-parenchymal strain gauge probes (Codman ICP MicroSensor; Codman & Shurtlef Inc., Raynham, MA, USA) placed in the frontal lobe or using external ventricular drains (Medtronic, Minneapolis, MN, USA). rSO_2_ of the left and right frontal lobes was measured using NIRS monitoring pads placed on the left and right forehead (Covidien INVOS 5100C) when possible.

Analogue-to-digital signal converters (Data Translations, DT9804 or DT9826) were utilized to capture ABP and ICP signals at a sampling frequency of 100 Hz. rSO_2_ was recorded from direct digital serial output from the monitoring device at a sampling frequency of 1 Hz. Intensive Care Monitoring (ICM+) software (Version 8.5, Cambridge Enterprise Ltd., Cambridge, UK) was utilized to store and time link the digitized physiologic signals.

Demographic data, including age, biologic sex, Marshal computed tomography (CT) score, admission Glasgow Coma Scale (GCS), admission pupil exam, and metabolic parameters, were collected for cohort characterization. Radiographic evidence of significant acute subdural hematomas, epidural hematomas, cerebral contusions, and scalp hematomas were also collected. This radiographic data were utilized to identify causes of interference in NIRS-based rSO_2_.

### 2.4. Data Cleaning and Processing

Utilizing ICM+ software high resolution physiologic, data were cleaned and processed. Artifact clearing was performed manually by qualified personnel without knowledge of patient factors or study aims. For each patient ABP and ICP, as well as right and left rSO_2_ signals, were passed through a 10-s, non-overlapping moving average filter. This is a standard practice to remove high-frequency signals unrelated to cerebrovascular reactivity and to focus on time scales related to cerebral vasomotion [22,23]. CPP was then derived as the difference between ABP and ICP.

Three continuous indices of CVR were then derived. PRx was derived as a minute-by-minute updating Pearson correlation between ICP and ABP over a 300 s window of paired 10-s mean values [24]. Additionally, COx and COx_a were derived similarly by calculating the correlation between rSO_2_ with CPP and ABP, respectively [15]. This was performed for both left and right rSO_2_ signals. Given that all three continuous indices of CVR were computed using Pearson correlation coefficients, they have a theoretical range from −1 to +1 with higher values being thought to be associated with a vasopassive state indicative of CVR dysfunction. These indices are known to be noisy and, therefore, it is often recommended by their developers to average over at least 30-min periods to improve signal-to-noise ratio [24,25]. Analysis was initially attempted with higher resolution signals (minute-by-minute and 5-min-by-5-min); however, even basic models uniformly failed to converge. This was likely attributable to the high degree of noise in these indices of CVR, making them not amenable to modeling at higher temporal resolutions. As such, a 30-min non-overlapping moving average filter was applied to all computed CVR indices. These 30-min-by-30-min data streams were then exported into comma separated value (.csv) files for further analysis. Analysis using hour-by-hour and 3-h-by-3-h data was also conducted with good model convergence. The 30-min-by-30-min data were selected over other time resolutions as they provided a good balance between improved signal-to-noise and high temporal resolution.

### 2.5. Statistical Data Analysis

#### 2.5.1. Overview

Further analysis was conducted utilising R statistical software (Version 4.2.2, R Foundation for Statistical Computing, Vienna, Austria) and leveraged using the following packages: *blandr*, *forecast*, *ggplot*, *nlme*, *tidyverse*, *tseries*, and *zoo*. To improve multithreaded computational performance, the default Basic Linear Algebra Subprograms (BLAS) and the Linear Algebra Package (LAPACK) were replaced with OpenBLAS (Version 0.3.23, Institute of Software, Chinese Academy of Sciences, Beijing, China). Any missing data were removed without interpolation. To focus on the acute phase of injury, the data were truncated to the first five days of recording where the brain was most vulnerable to ongoing injury [26]. Radiographic data were utilized to select the side of COx and COx_a used for analysis. The right side was utilized unless the presence of artifact producing extravascular blood was identified on the right. In that case, if the left side was free from extravascular blood, it was utilized instead. If neither side was free of extravascular blood, that patient’s data were excluded. Finally, due to the exploratory nature of the study, alpha was set to 0.05 without further correction for multiple comparisons.

#### 2.5.2. Data Exploration

For initial data exploration, density plots were created for all three CVR indices over the entire cohort utilizing the 30-min-by-30-min time resolution data. Further to this, three Bland–Altman plots were created to examine CVR parameter agreement as follows: PRx vs. COx, PRx vs. COx_a, and COx vs. COx_a. Bland–Altman plots are a means of visually representing agreement between two methods of measurement while also providing information about both fixed bias and proportional bias. 

#### 2.5.3. Linear Modeling of CVR

Linear modeling of PRx from COx and COx_a was attempted utilizing methodologies previously described in the high-resolution cerebral physiology literature [27]. Classical linear modelling has the requisite assumption that samples are collected in an independent fashion. The dataset presented here does not fulfill this assumption in two ways, both of which could lead to spurious statistical relationships in this inferential modeling if not accounted for.

First, there is a hierarchical structure to the data. That is, multiple datapoints have been collected from individual patients which means that random effects particular to each subject would not be accounted for in typical linear modeling. To account for the random effects experienced at the subject level, linear mixed effects (LME) modeling was employed. This methodology allows for the use of hierarchical, or nested data, accounting for the possible random effects that may modulate the relationship between the variables of interest between subjects. In this case, the variables of interest are measures of CVR.

Secondly, datapoints collected sequentially in time are likely to have some degree of temporal autocorrelation. Previous work has identified this to be the case for high-resolution cerebral physiologic data pertaining to CVR. The autocorrelative structure of this physiologic data can be accounted for by utilizing time-series modeling such as autoregressive moving average (ARMA) modeling methods as previously described [27]. In such modeling, past values of the variable in question can be utilized to predict future values. The order of the autoregressive (AR) and moving average (MA) components refer to the number of sequential past values incorporated into the model. These can be varied to optimize model performance while adhering to the principal of parsimony. The interested reader is directed to recent texts on ARMA modeling and time-series analysis [28,29,30,31].

In the setting of LME modeling, the effects of temporal autocorrelation can be accounted for by embedding the ARMA structure of the residuals into the model, thereby, as best as possible, accounting for both the hierarchical and autocorrelative structure of the data. Practically, this was accomplished by utilising the lme() function of the nmle package in R. LME models of PRx from both COx and COx_a were created with random effects allowed to modulate the slope and intercept of the model on a per subject basis. When it came to optimizing the embedded AR and MA structure, multiple models were created, varying the AR and MA order independently from 0 to 4. This was based on work examining the ARMA structure of PRx at various time resolutions and through examining the autocorrelation function (ACF) and partial autocorrelation function (PACF) plots of PRx over the cohort [32]. This resulted in a total of 25 variations in the PRx ~ COx model and the PRx ~ COx_a model. The optimal structure for each model was then selected based on their Akaike information criterion (AIC), Bayesian information criterion (BIC), and the Log Likelihood (LL). Model adequacy was determined through confirmation of the normal distribution of model residuals through density and QQ-plots. Additionally examination of the ACF and PACF plots of the residuals were examined to determine if the ARMA structure had been fully accounted for.

The statistical significance of COx and COx_a as a regressor in each corrected model was examined to evaluate the strength of the relationship between these modalities. To evaluate the ability of COx and COx_a to model PRx, the Pearson correlation coefficient between modeled PRx and measured PRx was calculated for both the selected PRx ~ COx and PRx ~COx_a models. Finally, to examine the agreement modeled and actual values of PRx, Bland–Altman plots were generated.

## 3. Results

### 3.1. Study Population

In total, 82 patients were included in this retrospective study of prospectively collected data. A total of 4545 h of physiologic data were utilized for the analysis with a median recording time of 51.8 h (IQR 19.5–92 h) per patient. A sample of the raw parameter recordings from a single patient can be seen in Figure S1 while a sample of the derived CVR metrics for that same period can be seen in Figure S2. The full summary of the physiologic and demographic parameters for the cohort can be found in Table 1.

### 3.2. Relationship between PRx, COx, and COx_a

**Figure 1:**
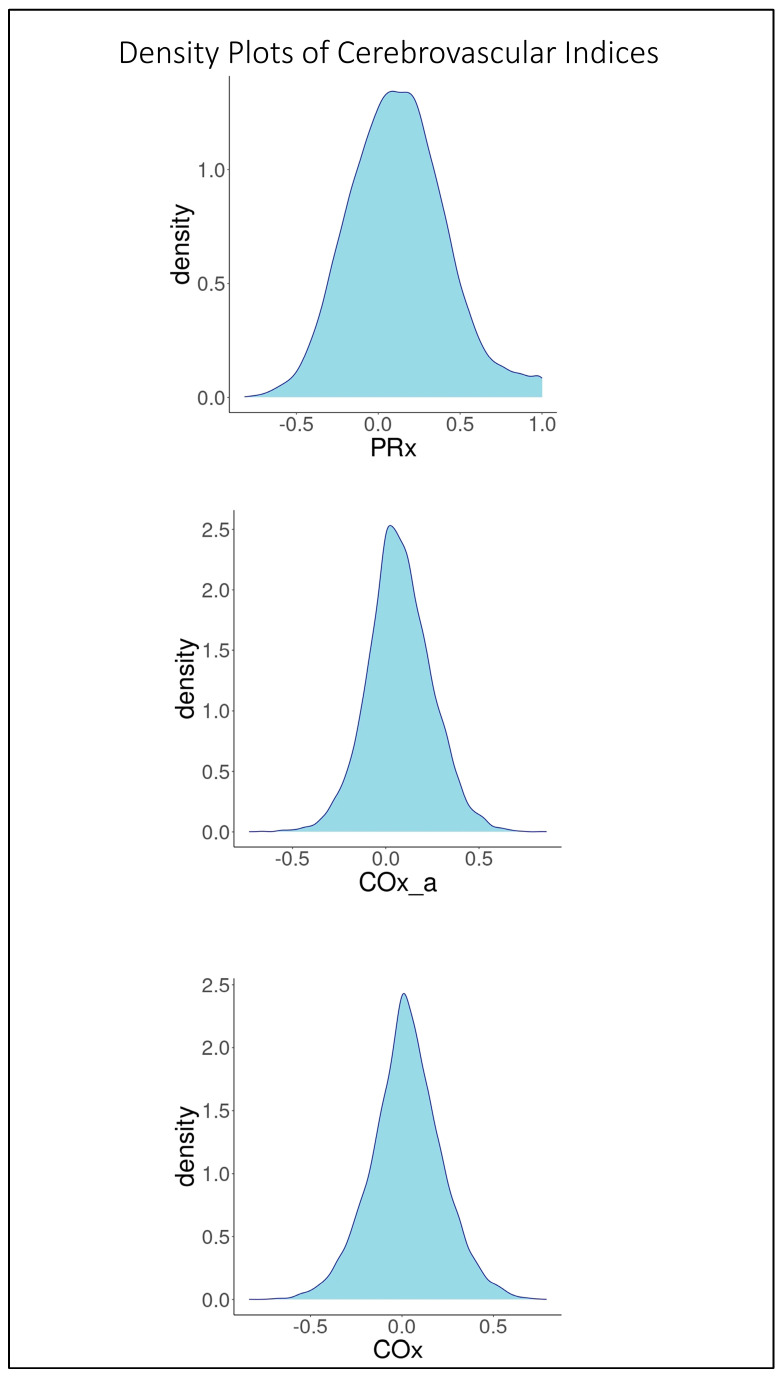
From top to bottom can be seen the density plots of the pressure reactivity index (PRx), the arterial blood pressure-based cerebral oxygen index (COx_a), and the cerebral perfusion pressure-based cerebral oxygen index (COx).

**Figure 2:**
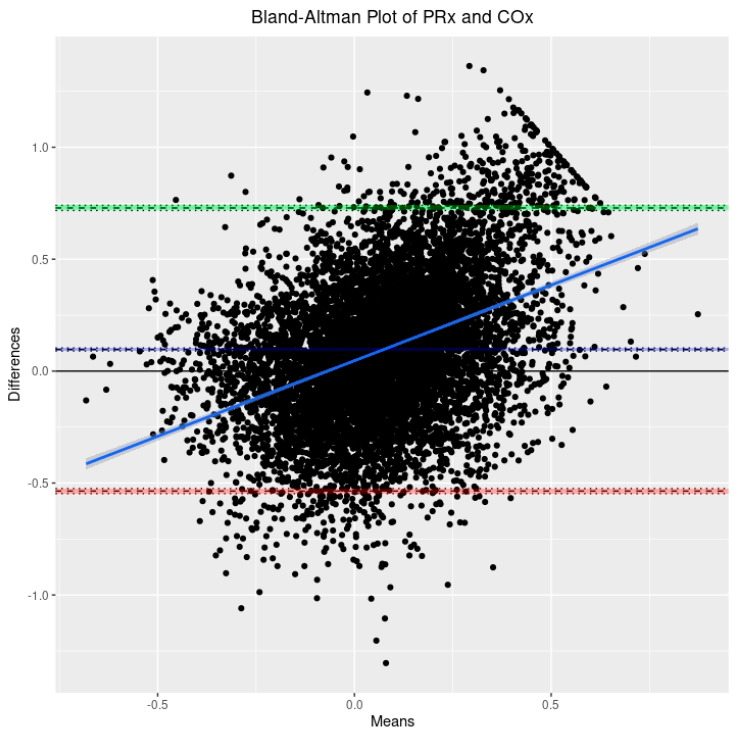
The Bland–Altman plot comparing the 30-min-by-30-min pressure reactivity index (PRx) and the cerebral perfusion pressure-based cerebral oxygen index (COx). The dashed blue line represents the mean difference between the two parameters, indicative of fixed bias. The green dashed line represents the +1.96 standard deviation above the mean while the red dashed line represents the −1.96 standard deviation below the mean. The solid blue line is the line of best fit, indicative of proportional bias. The 95% CI surround each line.

**Figure 3:**
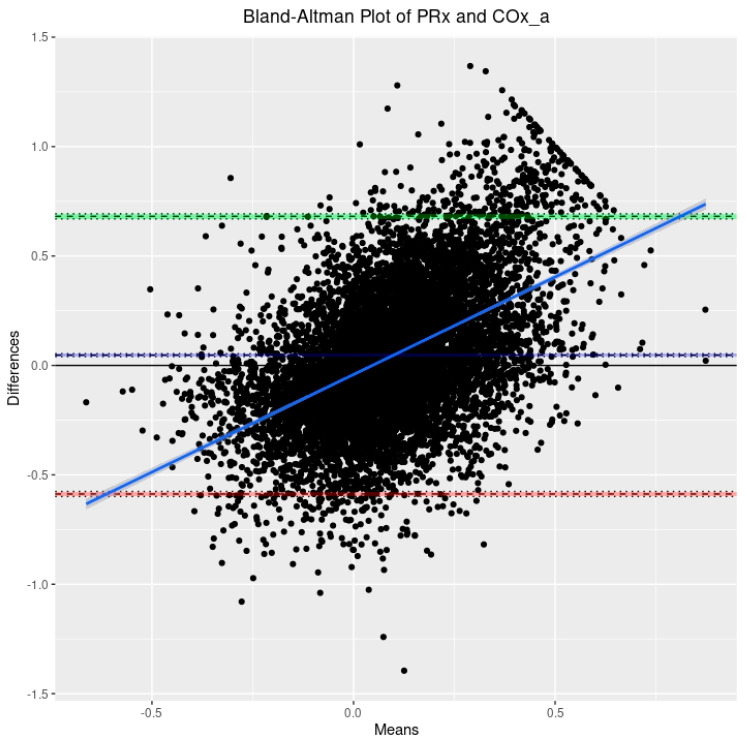
The Bland–Altman plot comparing the 30-min-by-30-min pressure reactivity index (PRx) and the arterial blood pressure-based cerebral oxygen index (COx_a). The dashed blue line represents the mean difference between the two parameters, indicative of fixed bias. The green dashed line represents the +1.96 standard deviation above the mean while the red dashed line represents the −1.96 standard deviation below the mean. The solid blue line is the line of best fit, indicative of proportional bias. The 95% CI surround each line.

**Figure 4:**
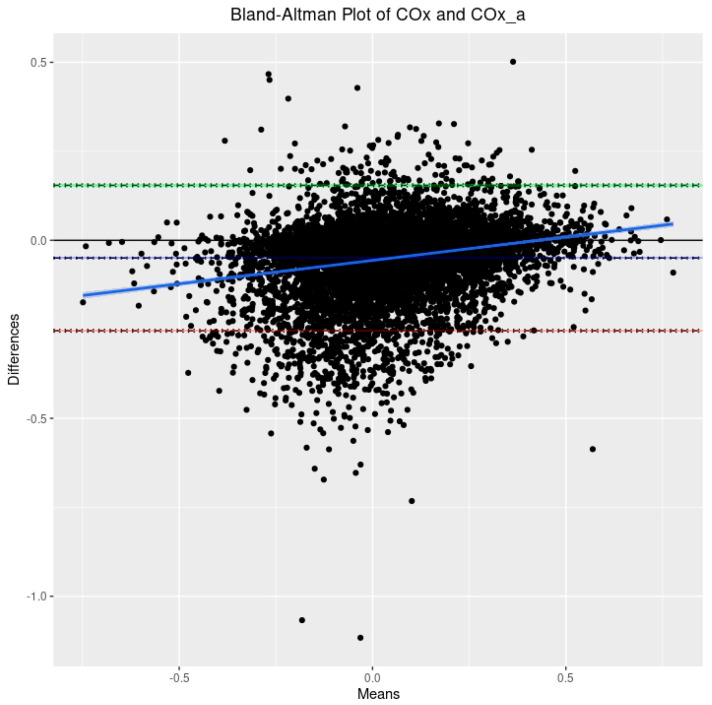
The Bland–Altman plot comparing the 30-min-by-30-min cerebral perfusion pressure-based cerebral oxygen index (COx) and the arterial blood pressure-based cerebral oxygen index (COx_a). The dashed blue line represents the mean difference between the two parameters, indicative of fixed bias. The green dashed line represents the +1.96 standard deviation above the mean while the red dashed line represents the −1.96 standard deviation below the mean. The solid blue line is the line of best fit, indicative of proportional bias. The 95% CI surround each line.

Based on the 9090 datapoints available in the cohort, the population-wide density plots of all three indices of CVR (30-min mean values) can be seen in Figure 1. PRx has a generally much larger distribution than COx or COx_a. Additionally, the PRx values tend to be higher in the cohort than in COx or COx_a. The Bland–Altman plots of 30 min mean raw PRx vs. COx, PRx vs. COx_a, and COx vs. COx_a are displayed in Figure 2, Figure 3 and Figure 4. For the plots of PRx vs. COx and PRx vs. COx_a, it can be seen that the limits of agreement are quite wide. There is also a fixed bias with PRx tending to be more positive in general than both the COx and COx_a values. Finally, there is also a proportional bias such so that COx and COx_a tend to be lower in magnitude than PRx at the extremes. 

### 3.3. LME Modeling of PRx Using COx and COx_a

Through varying the AR and MA order, 25 candidate LME models were created for both PRx ~ COx and PRx ~ COx_a. The various AIC, BIC, and LL values were then tabulated, as can be seen in Tables S1 and S2. These AIC, BIC, and LL values were utilized to identify which combinations of AR and MA order best accounted for the autocorrelative structure of that data. Ultimately, an AR order of 4 and an MA order of 1 for both the PRx ~ COx and PRx ~COx_a model was deemed to be superior. In the PRx ~ COx LME model with an AR order of 4 and an MA order of 1, COx was still determined to be a significant regressor (Coef: 0.16, SE = 0.03, *p*-value < 0.01) following correction of the hierarchical and autocorrelative structure of the data. This was also the case for the PRx ~ COx_a LME model with an AR order of 4 and an MA order of 1, where COx_a was noted to be a significant regressor (Coef: 0.17, SE = 0.03, *p*-value < 0.01). This indicates that COx and COx_a are related to PRx. Density plots and QQ-plots for the residuals of both selected models were inkeeping with normally distributed residuals indicative of model adequacy. This can be in seen in Figures S3 and S4. However, the PACF and ACF of both the COx- and COx_a-based models still had significant lags, indicating that the autocorrelative structure was not fully accounted for by the selected models. This can be seen in Figures S5–S8. 

**Figure 5:**
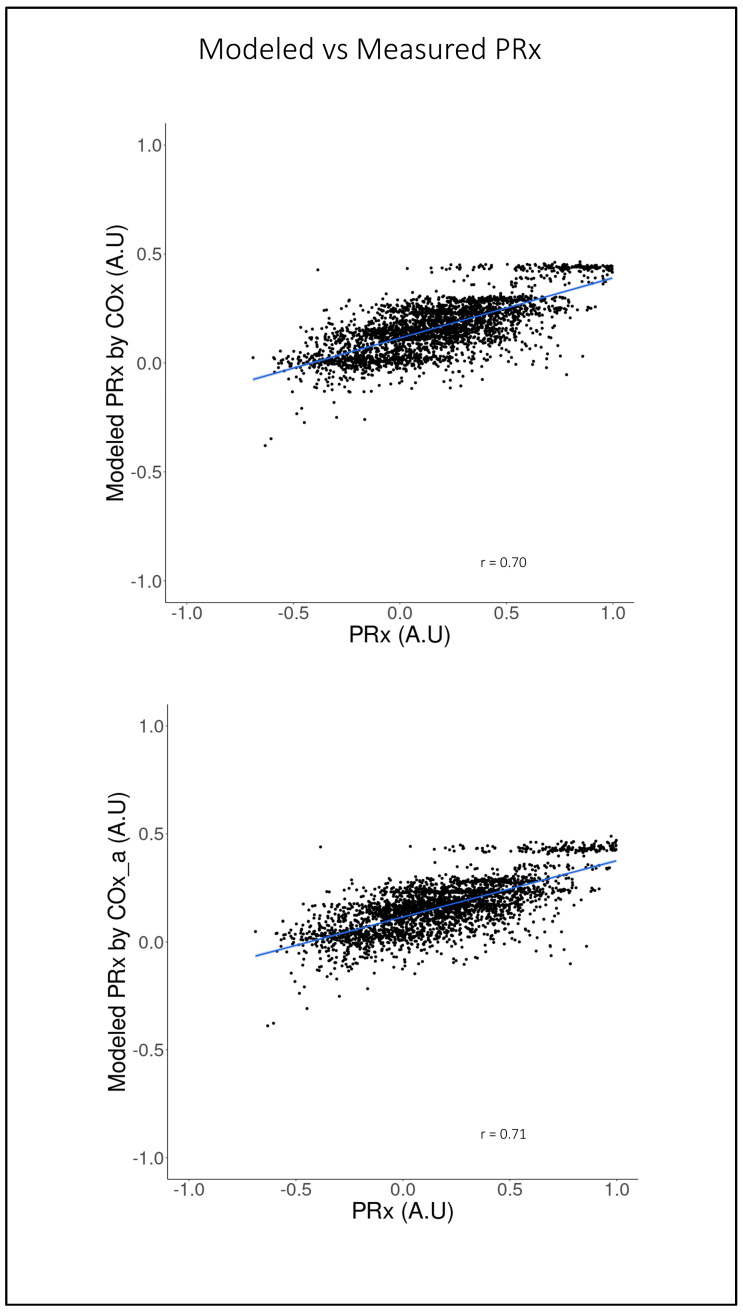
On the top is the modeled pressure reactivity index (PRx) by the cerebral perfusion pressure-based cerebral oxygen index (COx) versus the actual PRx. On the bottom is the modeled pressure reactivity index (PRx) by the arterial blood pressure-based cerebral oxygen index (COx_a) versus the actual PRx. Line of best fit in blue. All axes are in arbitrary units (AU).

**Figure 6:**
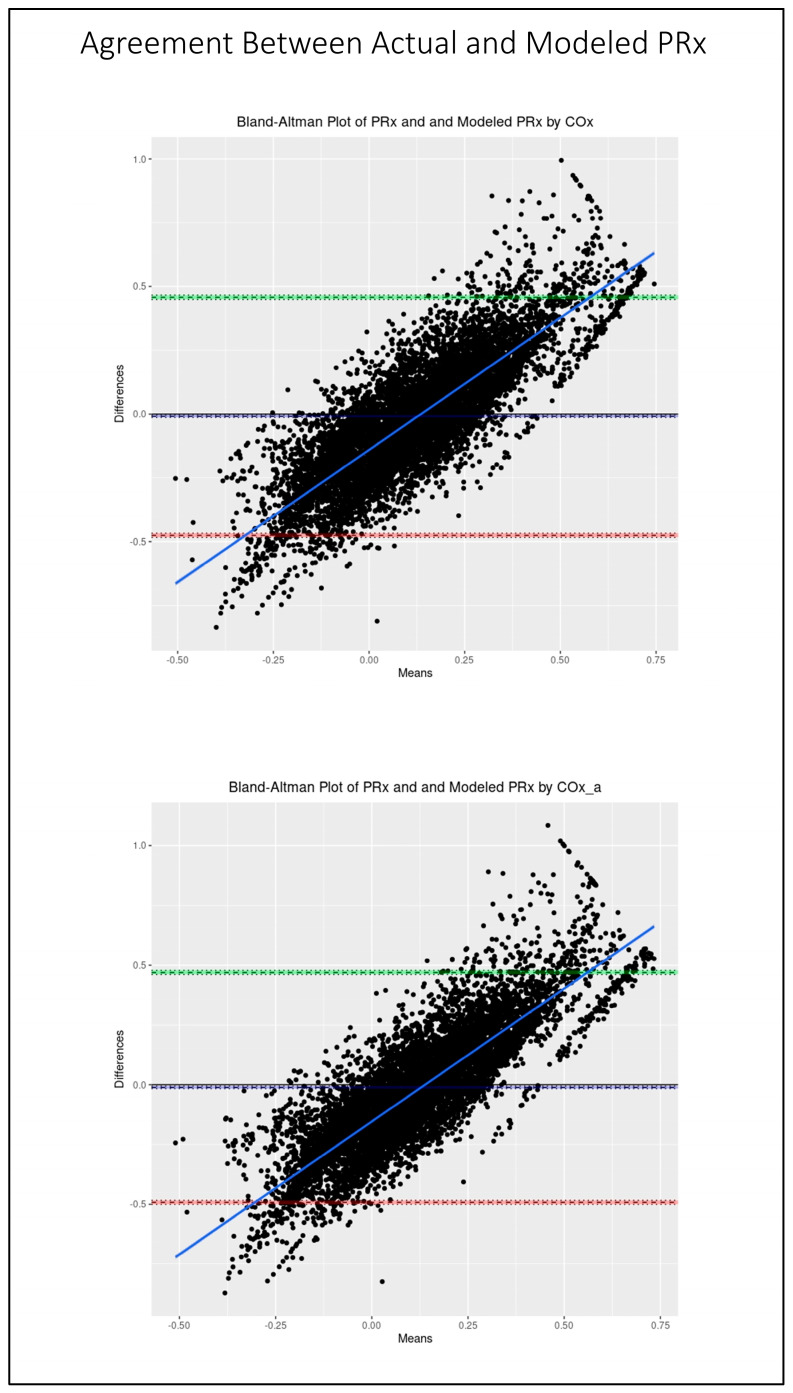
The Bland–Altman plots comparing actual pressure reactivity index (PRx) and modeled PRx by cerebral perfusion pressure-based cerebral oxygen index (COx), top, and by arterial blood pressure-based cerebral oxygen index (COx_a), bottom. The dashed blue line represents the mean difference between the two parameters, indicative of fixed bias. The green dashed line represents the +1.96 standard deviation above the mean while the red dashed line represents the −1.96 standard deviation below the mean. The solid blue line is the line of best fit, indicative of proportional bias. The 95% CI surround each line.

Figure 5 shows the scatter plots of the modeled PRx by COx and by COx_a vs. the actual PRx. As can be seen, there is good fit with strong correlation between the predicted and actual values of PRx for both the COx (r = 0.71, 95% CI: 0.70–0.73, *p* < 0.001)- and COx_a (r = 0.70, 95% CI: 0.68–0.72, *p* < 0.001)-based models. In Figure 6, the Bland–Altman plots for actual and modeled PRx can be see for both selected models. Notably, while there is minimal fixed bias, there is a proportional bias indicating that the modeled PRx tended to be of less magnitude than the actual PRx. Further the limits of agreement are quite wide and likely of clinical sgnificance, indicating suboptimal agreement between actual and modeled PRx.

## 4. Discussion

In this retrospective cohort study leveraging prospectively collected high-resolution physiologic data, a number of key insights can be garnered about rSO_2_-based indices of CVR and how they related to PRx. These insights help move CVR monitoring towards less invasive modalities with improved spatial resolution. First, it appears that the high degree of noise in indices of CVR present meaningful analysis at temporal resolutions of less than 30 min. This is based on the failure of models to converge when higher resolution data are utilized. This is inkeeping with the previous literature regarding these monitoring modalities, indicating that due to the significant noise in these parameters, the averaging of data over at least 30 min periods was recommended to improve signal-to-noise [24,25]. This may be seen as a disadvantage of the broader category of continuous measures of CVR when compared with neuroimaging-based methods that have inherently better signal-to-noise [3,33,34]. It should be noted that even with a temporal resolution of 30 min, continuous indices provide a much better temporal resolution than is practically achievable utilizing neuroimaging-based methods. Additionally, this is achieved at the bedside without the need for the manual manipulation of ABP, features that are desirable in a critically ill patient [3].

Next, PRx appears to be a much more broadly distributed index than COx or COx_a. Beyond this, there appears to be both a fixed bias, such that COx and COx_a tend to be absolutely less than PRx, and a proportional bias, which results in COx and COx_a tending to be of less magnitude at the extremes of CVR as measured by PRx. This is of significance because it indicates that COx or COx_a cannot serve as an absolute direct substitute for PRx when adopting previously established thresholds or critical values, as has been previously reported for PRx [9,35]. While the existing literature already demonstrates that COx and COx_a are able to detect the lower limit of autoregulation in large animal models [15,16], these critical thresholds will need to be reestablished in humans for COx and COx_a, distinct from the work performed to identify them in PRx.

Second, in this cohort, COx and COx_a were found to exhibit tight agreement with one another through Bland–Altman analysis. There is a slight fixed bias, with COx_a tending to be larger than COx, and a slight proportional bias, with the magnitude of COx_a tending to be less than that of COx. This is in agreement with recent work finding these parameters to cluster closely together in high-resolution multidimensional physiologic space [20]. This is significant as COx_a, which does not require invasive ICP monitoring, has the ability to be an entirely non-invasive means of continuously monitoring CVR [36]. This would open the door to CVR monitoring in a whole host of applications not currently available. Additionally, the knowledge that COx_a may further underestimate the extremes of CVR will help guide future studies leveraging its non-invasive nature.

Finally, even when the hierarchical and autocorrelative structure of the data were accounted for, both COx and COx_a were found to be significant regressors in linear models of PRx, with modeled PRx having a strong correlation with measured PRx. This indicates that while rSO_2_-based indices of CVR are not a drop-in replacement for PRx, they do contain significant information about PRx and, therefore, CVR. This strengthens the body of literature supporting the validity of these indices as measures of CVR. Coupled with the non-invasive nature of COx_a, CVR monitoring may then be expanded to include healthy populations where the monitoring of CVR may provide insights into physiologically normal processes. The monitoring of CVR could also be facilitated in pathologic states where ICP monitoring is not indicated. This could be in the setting of low-grade brain injuries, like concussion or systemic pathologies, that indirectly effect the brain, such as through sepsis. It could also be explored in the chronic phase of recovery from brain injury where the role of CVR is still unclear.

### 4.1. Limitiation

While this study benefits from a relatively large sample size, being the largest such study to date, to our knowledge, and rigorous statistical analysis, it does contain limitations that must be considered. First, the data originated from a single institution and are therefore subject to unaccounted for institution-specific practice patterns; although, as previously noted, contemporary Brain Trauma Foundation guideline-based management was followed [21]. Secondly, linear modeling was used to reduce complexity and was felt to be appropriate given the lack of prior evidence indicating that alternative modeling techniques may be more appropriate. However, this does mean that nuances in the relationship between rSO_2_-based indices of CVR and PRx might not have been fully captured. Further to this, the full autocorrelative structure of the data was not able to be fully accounted for by the selected models, indicating the need for possibly more complex time-series modeling. Additionally, the utilization of a single NIRS channel for analysis does mean that some information was lost. While other studies have previously averaged signals [25], there is no evidence to guide how these multiple channels should be handled. Finally, while LME modeling allows for the inferential modeling of the relationship between these indices of CVR, by its nature, it does not produce a model that can be transferred to other populations as it is specific to the cohort from which it was derived. Beyond this, the wide limits of agreements between the modeled and actual PRx are likely to not be clinically acceptable.

### 4.2. Future Directions

This study lays the groundwork for further investigation into rSO_2_-based CVR monitoring. This work can be expanded through the utilization of more complex modeling techniques to further characterize the relationship between these indices of CVR and PRx. Further, contemporary machine learning and artificial intelligence techniques may provide more accurate and generalizable models of PRx from COx and COx_a. These more sophisticated techniques may also be better positioned to incorporate multiple channels of NIRS data.

Beyond simply modeling PRx, the outcome association of COx and COx_a will also need to be further explored. If found to be associated with outcomes independently, their less invasive nature may allow for these indices to supplant PRx. Ultimately, outcome association will also lead to investigation into CVR, measured by COx or COx_a, as a possible modifiable therapeutic target.

Finally, the entirely non-invasive nature of COx_a should be taken advantage of to explore CVR in alternative settings. It can also be leveraged to examine the natural history of CVR into the chronic phase of neural injury.

## 5. Conclusions

In this retrospective cohort study of high-resolution physiological data, rSO_2_-based indices of CVR were found to strongly relate to one another and to be significant regressors in corrected linear models of PRx. This strengthens the body of evidence indicating that COx and COx_a may be appropriate alternatives to PRx for the measurement of CVR. Further work is needed to better characterize the relationship between these indices of CVR. This has the potential to open the door to continuous CVR monitoring in settings were ICP monitoring is not indicated.

## Figures and Tables

**Table 1:** Cohort (N = 82) Demographic and Physiologic Summary Statistics.

Demographic Parameter		Median (IQR) or N (%)
**Age**		42 (28.5–59.25)
**Sex**	**Male**	65 (79.3)
	**Female**	17 (20.7)
**Admission GCS**		6.5 (4–8)
**Follow-up GOSE at 6 Months**		6 (1–7)
**Admission Pupil Exam**	**Bilaterally Unreactive**	13 (15.9)
	**Unilaterally Unreactive**	16 (19.5)
	**Bilaterally Reactive**	53 (64.6)
**Admission Marshall CT Score**	**I**	0 (0.0)
	**II**	3 (3.7)
	**III**	23 (28.0)
	**IV**	15 (18.3)
	**V**	41 (50.0)
	**VI**	0 (0.0)
**Largest Lesion Type**	**SDH**	47 (57.3)
	**EDH**	4 (4.9)
	**Cerebral Contusion**	10 (12.2)
	**DAI**	6 (7.3)
	**tSAH**	15 (18.3)
**Surgical Intervention**	**Yes**	50 (61.0)
	**No**	32 (39.0)
**ICP monitoring method**	**Intraparenchymal Probe**	77 (93.9)
	**External Ventricular drains**	5 (6.1)
**Admission HgB (g/L)**		135 (113–147)
**Admission Serum Glucose (mmol/L)**		8.05 (7–10.95)
**Average PaO_2_ (mmHg) Over Course of Recording**		109 (87–138)
**Average PaCO_2_ (mmHg) Over Course of Recording**		37 (34–40)
**Average Blood Gas pH Over Course of Recording**		7.43 (7.39–7.47)
**Side of rSO_2_ Used**	**Right**	66 (80.5)
	**Left**	16 (19.5)
**Frontal Contusion Present**	**Right**	9 (11.0)
	**Left**	7 (8.5)
**Frontal Scalp Hematoma Present**	**Right**	7 (8.5)
	**Left**	6 (7.3)
**PRx**		0.11 (−0.08–0.31)
**COx**		0.02 (−0.09–0.15)
**COx_a**		0.07 (−0.03–0.18)

COx = Cerebral Perfusion Pressure-Based Cerebral Oxygen Index, COx_a = Arterial Blood Pressure-Based Cerebral Oxygen Index, CT = Computed Tomography, DAI = Diffuse Axonal Injury, EDH = Epidural Hematoma, GCS = Glasgow Coma Scale, GOSE = Extended Glasgow Outcome Scale, HgB = Hemoglobin, ICP = Intracranial Pressure, IQR = Interquartile Range, N = Number of Subjects, PaCO_2_ = Partial Pressure of Carbon Dioxide in Arterial Blood, PaO_2_ = Partial Pressure of Oxygen in Arterial Blood, PRx = Pressure Reactivity Index, rSO_2_ = Regional Cerebral Oxygen Saturation, SDH = Subdural Hematoma. tSAH = Traumatic Subarachnoid Hemorrhage.

## Data Availability

The raw data supporting the conclusions of this article will be made available by the authors, without undue reservation.

## Supplementary Materials

The following supporting information can be downloaded at: https://www.mdpi.com/article/10.3390/s24020499/s1, Table S1: COx-based model selection, Table S2: COx_a-based model selection, Figure S1: Sample raw parameter recordings, Figure S2: Sample-derived CVR metrics, Figure S3: Normality test of COx-based model residuals, Figure S4: Normality test of COx-based model residuals, Figure S5: ACF plot of COx-based model residuals, Figure S6: PACF plot of COx-based model residuals, Figure S7: ACF plot of COx_a-based model residuals, Figure S8: PACF plot of COx_a-based model residuals.
