# Supplementary material for: Non-Invasive Estimation of Intracranial Pressure-Derived Cerebrovascular Reactivity Using Near-Infrared Spectroscopy Sensor Technology in Acute Neural Injury: A Time-Series Analysis
Source: Sensors (Basel). 2024 Jan 13;24(2):499. doi: 10.3390/s24020499 (PMC10818714; doi:10.3390/s24020499)
Supplement: Supplementary file 1 [file sensors-24-00499-s001.zip › sensors-2778622-supplementary.pdf]

## Supplementary Material

**Table S1: Presented are the Akaike Information Criterion (AIC), Bayesian Information Criterion, and Log Likelihood (LL) for the various linear mixed effects models of the pressure reactivity index (PRx) modeled from the cerebral perfusion pressure based cerebral oxygen index (COx). For each model, the autoregressive (AR) and moving average (MA) order of the correction for autocorrelated residuals was varied from 0 to 4. The top four performing models by each criterion are in bold.**

| AR Order | MA Order | AIC | BIC | LL |
|---|---|---|---|---|
| 0 | 0 | -1564.486 | -1521.796 | 788.2428 |
| 0 | 1 | -3674.154 | -3624.349 | 1844.077 |
| 0 | 2 | -4349.61 | -4292.69 | 2182.805 |
| 0 | 3 | -4704.492 | -4640.458 | 2361.246 |
| 0 | 4 | -4922.298 | -4851.148 | 2471.149 |
| 1 | 0 | -4869.732 | -4819.928 | 2441.866 |
| 1 | 1 | -5502.863 | -5445.943 | 2759.431 |
| 1 | 2 | -5606.317 | -5542.282 | 2812.158 |
| 1 | 3 | -5619.35 | -5548.2 | 2819.675 |
| 1 | 4 | -5451.408 | -5373.144 | 2736.704 |
| 2 | 0 | -5233.401 | -5176.482 | 2624.701 |
| 2 | 1 | -5623.885 | **-5559.851** | 2820.942 |
| 2 | 2 | **-5632.101** | **-5560.952** | 2826.05 |
| 2 | 3 | -5610.333 | -5532.068 | 2816.166 |
| 2 | 4 | -5620.928 | -5535.549 | 2822.464 |
| 3 | 0 | -5403.569 | -5339.534 | 2710.784 |
| 3 | 1 | -5628.959 | **-5557.81** | 2824.48 |
| 3 | 2 | -5622.509 | -5544.245 | 2822.255 |
| 3 | 3 | -5628.568 | -5543.188 | **2826.284** |
| 3 | 4 | -5625.409 | -5532.915 | 2825.705 |
| 4 | 0 | -5479.987 | -5408.838 | 2749.994 |
| 4 | 1 | **-5633.172** | **-5554.908** | **2827.586** |
| 4 | 2 | -5625.643 | -5540.263 | 2824.821 |
| 4 | 3 | **-5633.732** | -5541.238 | **2829.866** |
| 4 | 4 | **-5633.162** | -5533.553 | **2830.581** |

**Table S2: Presented are the Akaike Information Criterion (AIC), Bayesian Information Criterion, and Log Likelihood (LL) for the various linear mixed effects models of the pressure reactivity index (PRx) modeled from the arterial blood pressure based cerebral oxygen index (COx_a). For each model, the autoregressive (AR) and moving average (MA) order of the correction for autocorrelated residuals was varied from 0 to 4. The top four performing models by each criterion are in bold.**

| AR Order | MA Order | AIC | BIC | LL |
|---|---|---|---|---|
| 0 | 0 | -1317.431 | -1274.742 | 664.7157 |
| 0 | 1 | -3551.745 | -3501.94 | 1782.872 |
| 0 | 2 | -4259.803 | -4202.883 | 2137.901 |
| 0 | 3 | -4631.583 | -4567.549 | 2324.791 |
| 0 | 4 | -4868.566 | -4797.417 | 2444.283 |
| 1 | 0 | -4836.814 | -4787.009 | 2425.407 |
| 1 | 1 | -5476.539 | -5419.619 | 2746.269 |
| 1 | 2 | -5587.059 | -5523.025 | 2802.53 |
| 1 | 3 | -5599.298 | -5528.148 | 2809.649 |
| 1 | 4 | -5599.837 | -5521.573 | 2810.919 |
| 2 | 0 | -5201.64 | -5144.72 | 2608.82 |
| 2 | 1 | -5605.029 | **-5540.995** | 2811.515 |
| 2 | 2 | **-5612.489** | **-5541.34** | 2816.245 |
| 2 | 3 | -5594.489 | -5516.225 | 2808.244 |
| 2 | 4 | -5600.492 | -5515.113 | 2812.246 |
| 3 | 0 | -5380.567 | -5316.532 | 2699.283 |
| 3 | 1 | -5609.273 | **-5538.123** | 2814.636 |
| 3 | 2 | -5604.8 | -5526.536 | 2813.4 |
| 3 | 3 | -5609.565 | -5524.186 | 2816.783 |
| 3 | 4 | -5607.712 | -5515.218 | **2816.856** |
| 4 | 0 | -5460.73 | -5389.581 | 2740.365 |
| 4 | 1 | **-5614.835** | **-5536.57** | **2818.417** |
| 4 | 2 | -5606.785 | -5521.406 | 2815.393 |
| 4 | 3 | **-5616.421** | -5523.927 | **2821.21** |
| 4 | 4 | **-5616.213** | -5516.604 | **2822.106** |

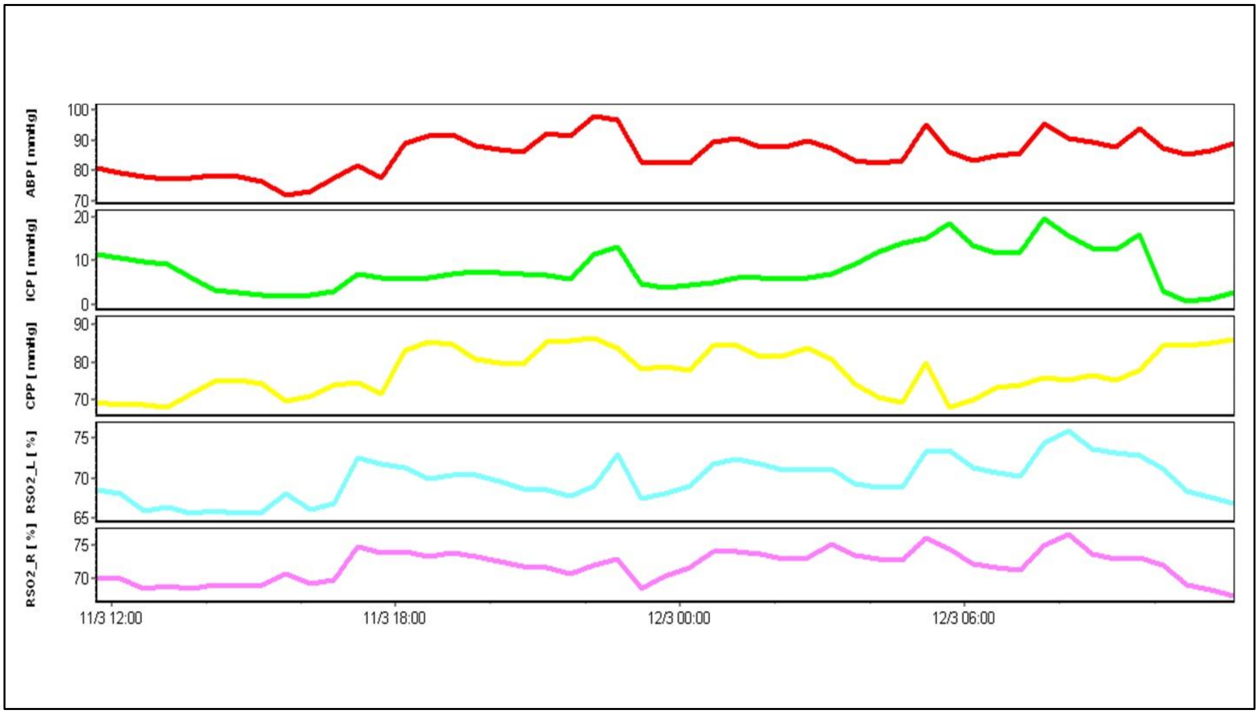


**Figure S1: Sample 30-minute-by-30-minute of the raw data parameters arterial blood pressure (ABP), intracranial pressure (ICP), cerebral perfusion pressure (CPP), regional cerebral oxygen saturation on the left (RS02_L) and right (RS02_R) over a 24 hour period for a single patient recorded with ICM+ software.**

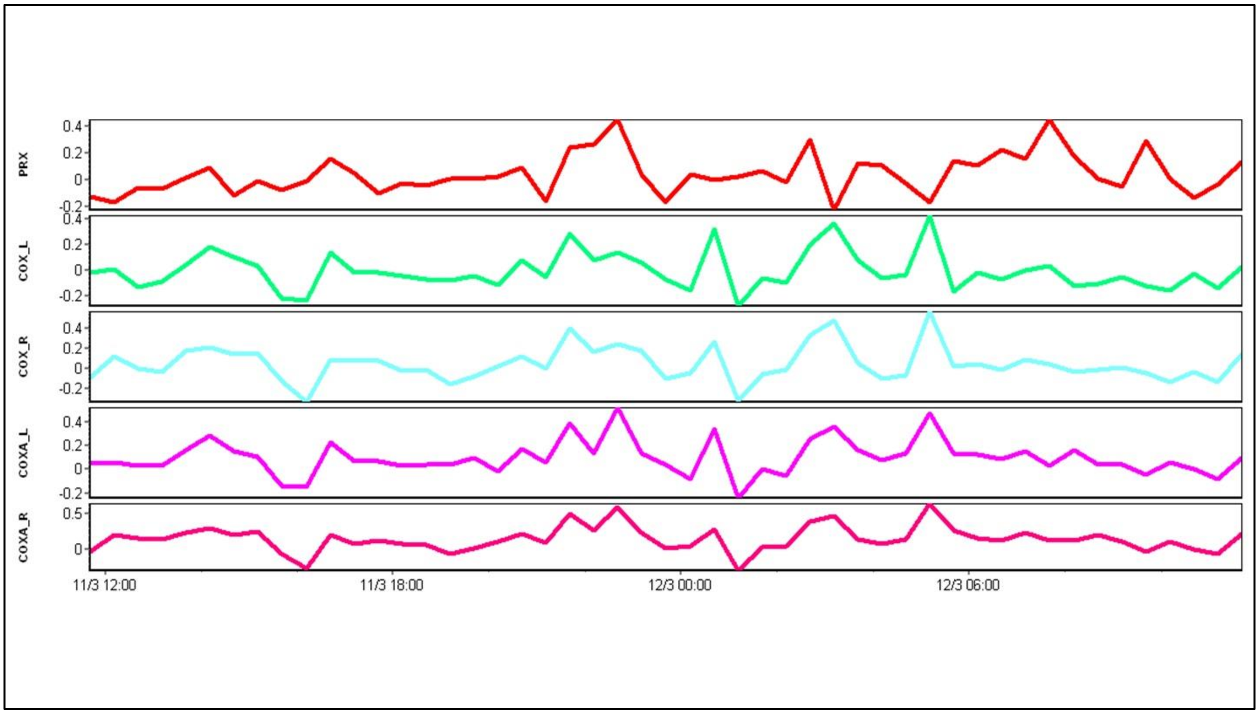


**Figure S2: Sample 30-minute-by-30-minute of the cerebrovascular reactivity metrics pressure reactivity index (PRx), cerebral perfusion pressure based cerebral oxygen index on the left (COX_L) and right (COX_R), as well as the arterial blood pressure based cerebral oxygen index on the left (COXA_L) and right (COXA_R) over a 24 hour period for a single patient calculated with ICM+ software.**

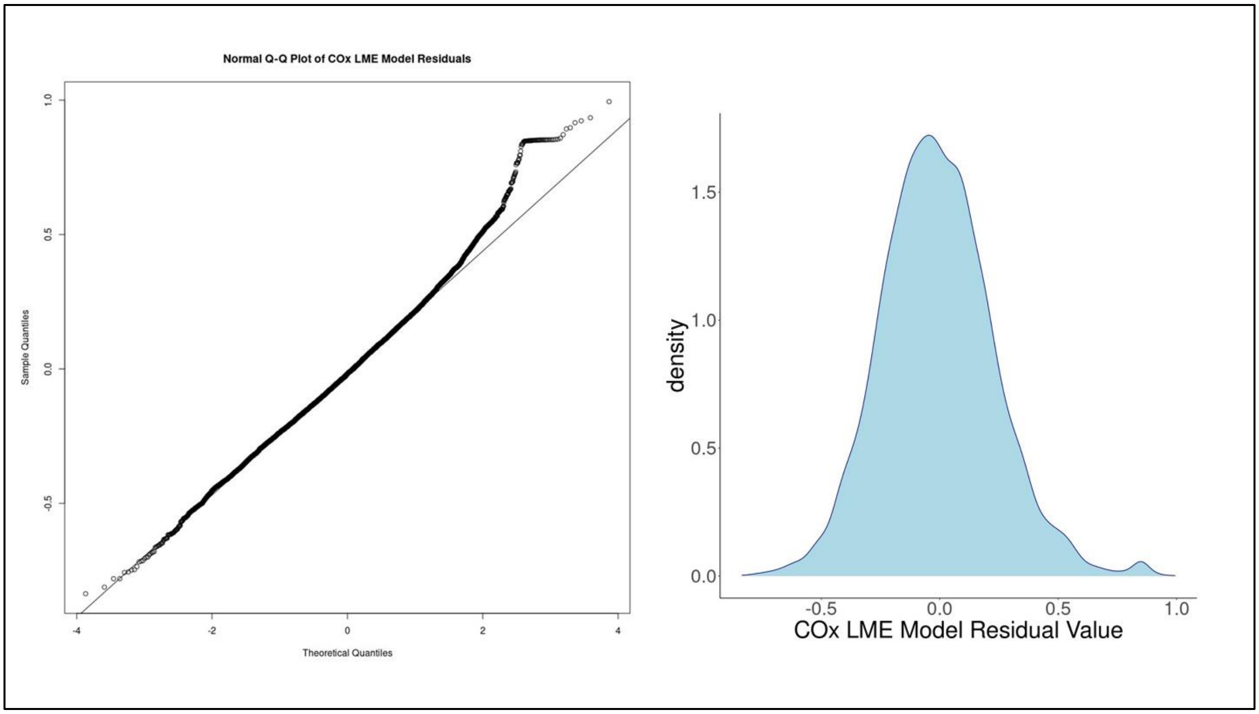


**Figure S3: On the right is presented the density plot of the residuals of the selected linear mixed effects (LME) model with autoregressive order 4 and moving average order 1 of the pressure reactivity index (PRx) based on the cerebral perfusion pressure based cerebral oxygen index (COx). The Q-Q plot of the residuals in on the left. Both are consistent with normally distributed residuals.**

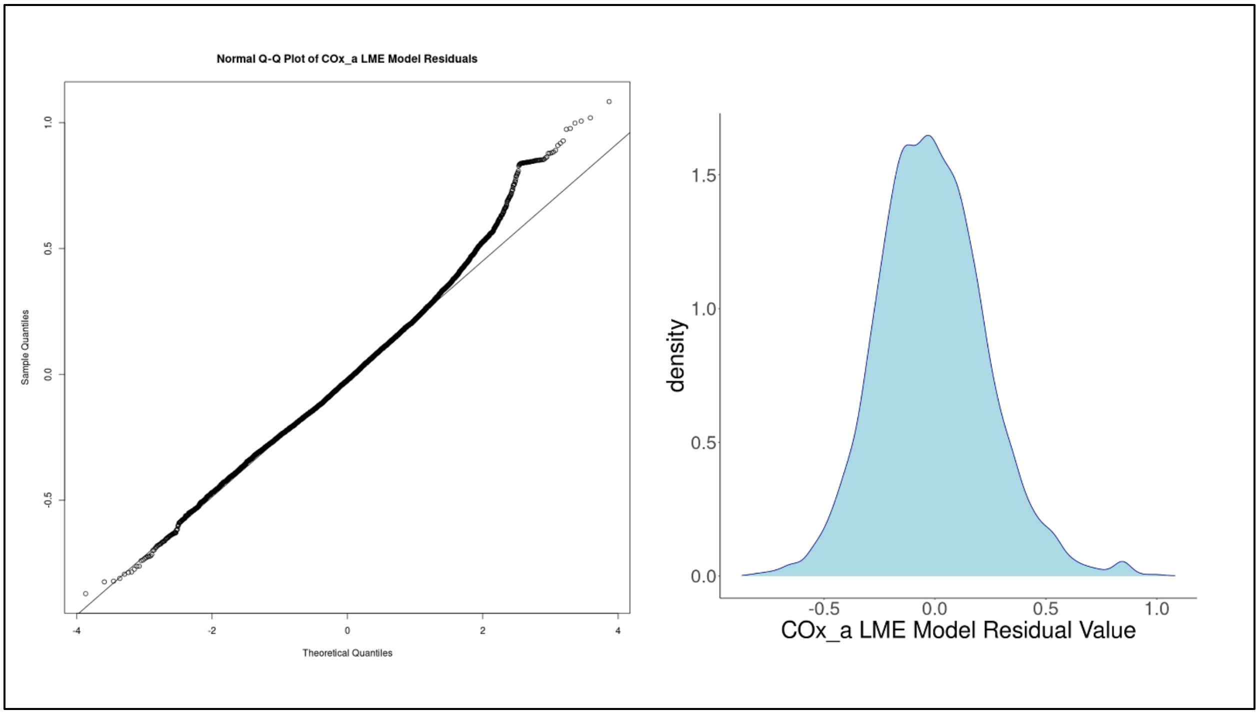


**Figure S4: On the right is presented the density plot of the residuals of the selected linear mixed effects (LME) model with autoregressive order 4 and moving average order 1 of the pressure reactivity index (PRx) based on the arterial blood pressure based cerebral oxygen index (COx_a). The Q-Q plot of the residuals in on the left. Both are consistent with normally distributed residuals.**

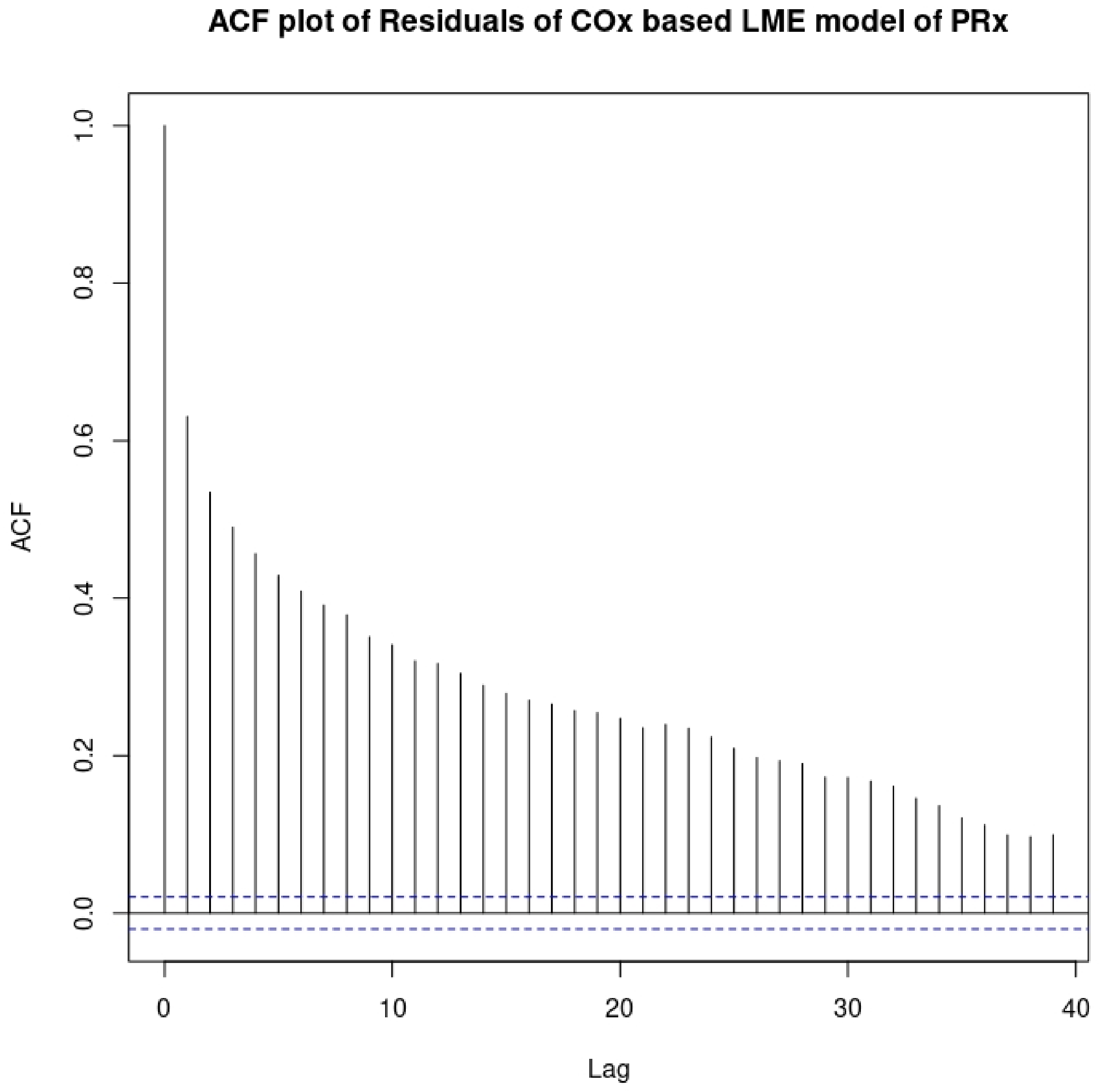


**Figure S5: The autocorrelative function (ACF) plot of the residuals of the selected linear mixed effects model (LME) of PRx based on COx. The dashed blue line represents the significance levels which were set to a correlation level of +/-(2/N1/2), where N is the number of samples.**

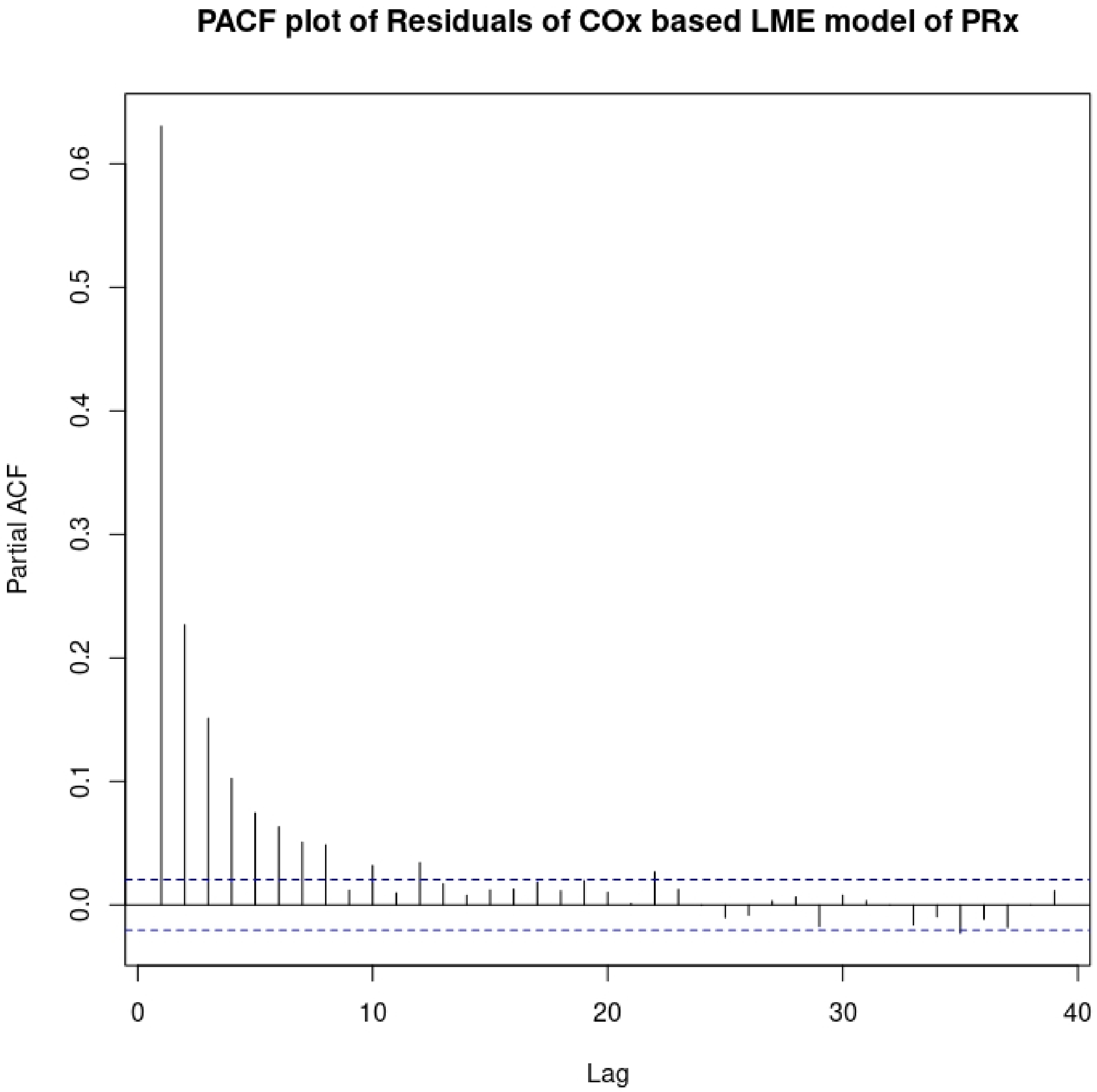


**Figure S6: The partial autocorrelative function (PACF) plot of the residuals of the selected linear mixed effects model (LME) of PRx based on COx. The dashed blue line represents the significance levels which were set to a correlation level of +/-(2/N1/2), where N is the number of samples.**

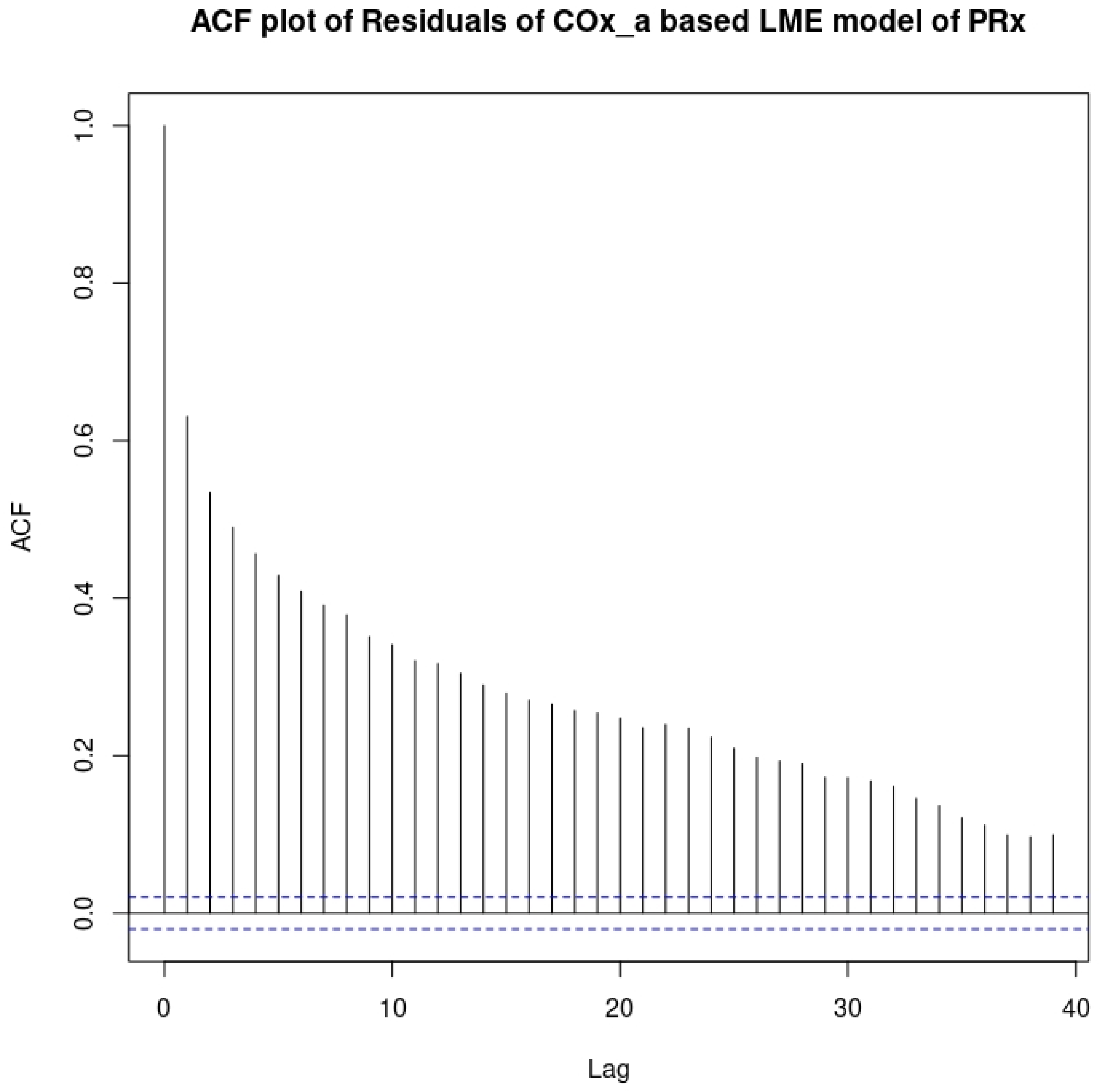


**Figure S7: The autocorrelative function (ACF) plot of the residuals of the selected linear mixed effects model (LME) of PRx based on COx_a. The dashed blue line represents the significance levels which were set to a correlation level of +/-(2/N1/2), where N is the number of samples.**

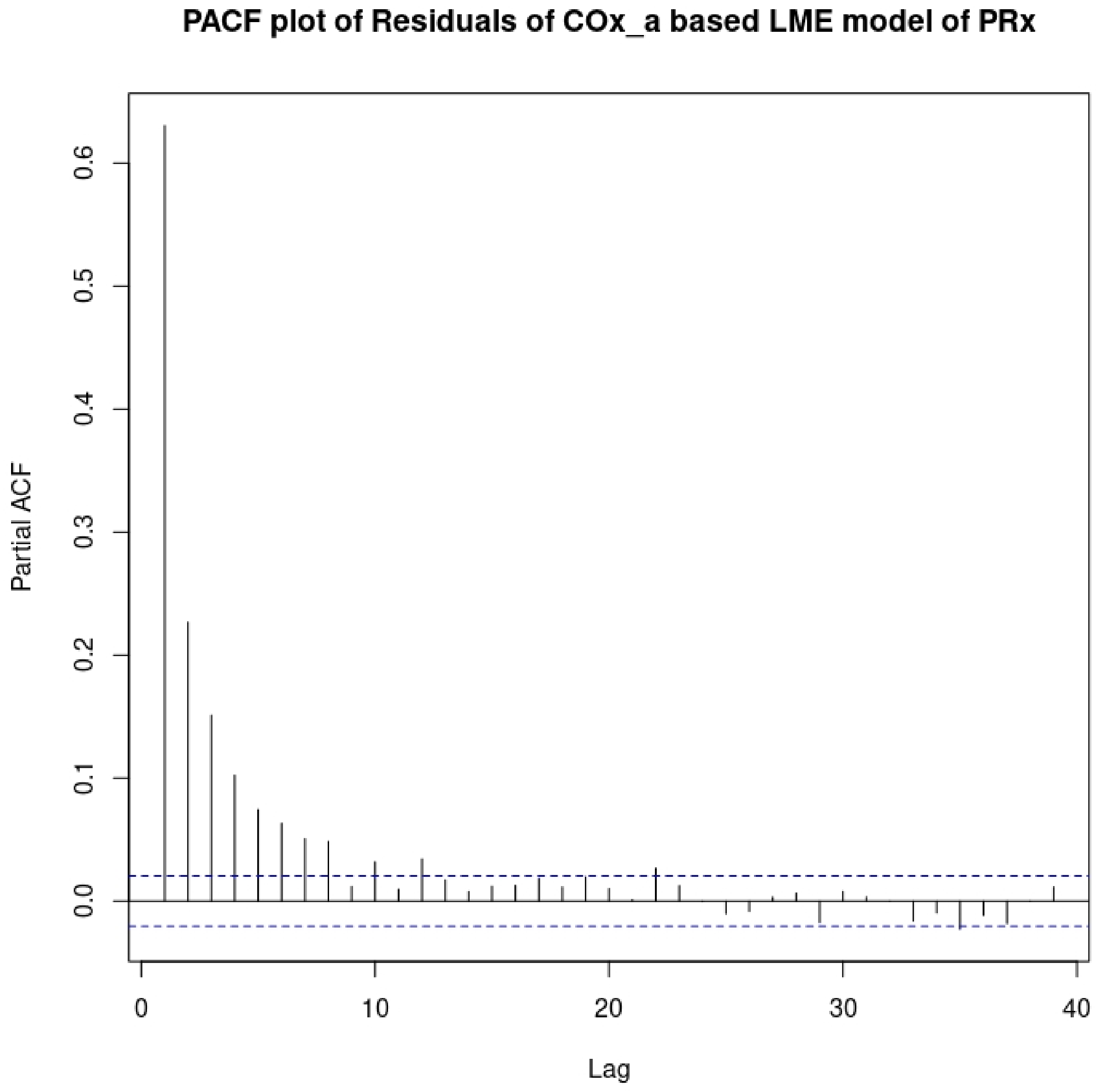


**Figure S8: The partial autocorrelative function (ACF) plot of the residuals of the selected linear mixed effects model (LME) of PRx based on COx_a. The dashed blue line represents the significance levels which were set to a correlation level of +/-(2/N1/2), where N is the number of samples.**
